# Sexual Orientation and Personality Dimensions Measured by the Junior Temperament and Character Inventory among Adolescents

**DOI:** 10.1007/s10508-025-03180-9

**Published:** 2025-07-03

**Authors:** Therése Skoog, Emma Claesdotter-Knutsson, Anders Håkansson, Sabina Kapetanovic, Arne Gerdner

**Affiliations:** 1 Department of Psychology, University of Gothenburg, 405 30 Gothenburg, Sweden; 2 Department of Clinical Sciences, Lund, Faculty of Medicine, Lund University, Lund, Sweden; 3 Department of Social and Behavioural Studies, University West, Trollhättan, Sweden; 4 School of Health and Welfare, Jönköping University, Jönköping, Sweden

**Keywords:** Sexual orientation, Personality, Temperament, Character, Adolescents, Junior Temperament and Character Inventory

## Abstract

Research on adolescent sexual orientation has expanded in recent decades, yet little is known about its associations with personality traits during this developmental period. This study examined personality dimensions, as measured by the Junior Temperament and Character Inventory, across adolescent sexual orientation groups (asexual, homosexual, heterosexual, bisexual) in a community-based sample of Swedish adolescents (*N* = 1195). Significant differences emerged primarily among girls, with bisexual girls exhibiting higher novelty seeking and harm avoidance but lower reward dependence and self-directedness compared to heterosexual peers. Asexual girls displayed lower novelty seeking than all other groups. Among boys, bisexual adolescents showed elevated harm avoidance relative to their heterosexual and asexual peers. Overall, effect sizes were small, though some post hoc comparisons yielded moderate to large effects. These findings suggest that sexual orientation is linked to personality traits during adolescence, with notable gender differences in the nature and magnitude of these associations.

## Introduction

Adolescence is a time of sexual development and maturation, driven in large part by puberty (Olmstead, 2020; Skoog, 2023). One of the core aspects of adolescent sexual development is sexual orientation, which refers to “relative sexual attraction to men, to women, or to both" (Bailey et al., 2016, p. 45). A recent meta-analysis found that the average age of becoming aware of own non-heterosexual attraction is 12.7 years, marking the first of several sexual behavior milestones (Hall et al., 2021). Other milestones, such as first sexual activity, occur many years later, with the mean age being 18.1 years. By age 17, attraction patterns tend to stabilize (Claesdotter-Knutsson et al., in press). Consequently, attraction is an early indicator of youth sexual orientation and has been identified as the preferred measure in studies of adolescent sexual orientation (Austin et al., 2007).

The most commonly identified sexual orientations are asexual (no sexual and emotional attraction to any gender), homosexual (sexual and emotional attraction to the same gender), bisexual (sexual and emotional attraction to both/several genders), and heterosexual (sexual and emotional attraction to the opposite gender). Using sexual (and emotional) attraction as the measure of sexual orientation, prevalence estimates for these four orientations in late adolescence range between 0–23% (asexual), 3–11% (homosexual), 8–26% (bisexual), and 67–89% (heterosexual) (Beaver et al., 2016; Claesdotter-Knutsson et al., in press; Priebe & Svedin, 2013; Rahman et al., 2020). Generally, girls are more likely to report non-heterosexual attraction compared to boys, especially some degree of bisexual attraction. Whereas the body of research on sexual orientations among young people is growing and examines the role of sexual orientation in a wide range of psychological and social outcomes (e.g., Argyriou et al., 2020; Plöderl & Tremblay, 2015; Ross et al., 2018; Saewyc, 2011), studies on the associations between adolescent sexual orientation and personality remain scarce. The present study was designed to overcome this gap by exploring the association between sexual orientation and personality in a community sample of Swedish adolescents.

Personality refers to individual differences in the characteristic way people feel, think and behave (Soto & Tackett, 2015). By late adolescence, personality traits become more stable compared to earlier stages of development (Klimstra et al., 2009). These traits play a pivotal role in shaping the trajectory of individuals' lives and are intricately linked to important biological, psychological, social, and health outcomes during adolescence (Soto & Tackett, 2015). Links between sexual orientation and personality were studied as early as the 1930s (Lippa, 2005), and a modern meta-analysis of adult samples (Allen & Robson, 2020) has established associations between sexual orientation and personality in Big-5 personality model (i.e., neuroticism, openness, extraversion, agreeableness, and conscientiousness; Costa & McCrae, 1992). Specifically, research findings indicate that homosexual men generally exhibit higher levels of neuroticism compared to their heterosexual counterparts, while homosexual women tend to display lower levels of neuroticism in comparison to heterosexual women. Additionally, homosexual and bisexual individuals of both sexes have been found to score higher on openness to experience compared to their heterosexual counterparts. Finally, existing research has shown that bisexual men and women tend to exhibit lower conscientiousness than either heterosexual or homosexual individuals. Several mechanisms may underlie and account for links between sexual orientation and personality in youth. First, both sexual orientation and personality have been found to have partial biological or hereditary bases (Wang et al., 2019; Zwir et al., 2021). Consequently, shared biological or genetic influences may drive the development of both sexual orientation and personality traits (Luoto, 2020; Zietsch et al., 2011). Second, socialization processes and environmental factors have been argued to influence both sexual orientation and personality development (Wang et al., 2019; Zwir et al., 2021), which may also help explain the link. However, it should be noted that the extant empirical literature has not yet established that socialization impacts sexual attraction relative to gender (Bailey et al., 2016). From a social-environmental perspective, discrimination, stigma, and stress related to one’s sexual orientation could impact personality development, particularly in young people whose personalities are still evolving (Klimstra et al., 2009). Moreover, a recent study found that childhood experiences (adversity) is linked to sexual behavior including sexual orientation later in life, with sexual reward sensitivity as the suggested mechanism (Alley et al., 2024). Finally, from a behavioral perspective, it has been suggested that because sexuality and sexual orientation involve distinct behaviors, and behaviors are intertwined with personality traits, there may be connections between personality traits and the diverse behavioral expressions of sexuality, including sexual orientation (Jonason & Luoto, 2021).

The gender-shift theory proposes that gay men are more likely to exhibit traits traditionally associated with femininity, while lesbian women are more likely to exhibit traits traditionally associated with masculinity (Lippa, 2005). This shift is particularly evident in neuroticism, where homosexual men are predicted to score higher than heterosexual men (as women generally score higher than men), whereas homosexual women are expected to score lower than heterosexual women. Empirical support for this pattern in neuroticism has been provided by Allen and Robson (2020). Additionally, research indicates that bisexual individuals may have personality traits that fall between those of heterosexual and homosexual individuals in terms of gender typicality (Lippa, 2020). While Semenyna et al. (2024) examined other personality traits in relation to sexual orientation, their findings also contribute to understanding the broader personality differences among sexual orientation groups.

In contrast to the substantial literature on sexual orientation and personality among adults (for reviews, see Allen & Roson, 2020; Lippa, 2020), very few studies have been conducted with adolescent samples. This is problematic given that personality is thought to play an important role in adolescent sexuality (Harris et al., 2023; Kar et al., 2015) and that adolescence is a unique developmental period. Our review of the field identified only two relevant studies. In a longitudinal sample of early adolescents in Australia, Allen and Walter (2018) used the school aged temperament inventory and found that personality traits (neuroticism and conscientiousness) at age 12 predicted same-sex attraction two years later. In a cross-sectional study of 10,000 17-year-olds in the UK, Xu et al. (2022) examined associations between one aspect of personality (neuroticism) and sexual orientation, finding higher levels of neuroticism among bisexual and homosexual adolescents compared to heterosexual adolescents. These studies suggest that the association between sexual orientation and personality may already be present in adolescence, but further research is needed to confirm these findings.

### Current Knowledge Gap

Few studies have explored associations between sexual orientation and personality in adolescent samples (Allen & Walter, 2018; Xu et al., 2022). None, however, has examined this association using temperament and character traits underlying personality. This study aims to address this gap by exploring and comparing personality dimensions across sexual orientation groups within a large community-based sample of Swedish adolescents.

Given that associations between sexual orientation and personality are expected to occur on the biobehavioral level (Jonason & Luoto, 2021), we used the well-established Junior Temperament and Character Inventory (J-TCI, Boson et al., 2018; Luby et al., 1999) to measure adolescent personality. The TCI is a comprehensive psychobiological inventory of personality (Cloninger et al., 1993), which includes seven basic dimensions of temperament and character. According to the theory (Cloninger, 2004), temperament pertains to individual differences in emotional reactions that are relatively automatic responses to experiences, believed to have a substantial degree of heritability, and that remain stable throughout an individual's lifespan. The four measured dimensions of temperament in J-TCI are novelty seeking (tendency to seek out new and exciting experiences), harm avoidance (level of aversion to risk and harm), reward dependence (sensitivity to rewards and dependence on approval and positive feedback from others), and persistence (ability to sustain behavior in the face of frustration and fatigue).

In Cloninger’s (2004) theory, character concerns individual differences in self-concept, as well as variations in goals and values, all of which subsequently influence voluntary decisions, intentions, and the significance an individual attributes to their life experiences. The three aspects of character are self-directedness (ability to regulate and adapt behavior based on own values and goals), cooperativeness (capacity to cooperate with others, be empathetic, and maintain harmonious social relationships), and self-transcendence (ability to transcend self-centeredness and connect with broader spiritual or transcendent values).

Conceptually, the seven TCI dimensions converge with the Big-5 traits (Costa & McCrae, 1992) in several respects. At the same time, there are notable differences. Novelty seeking can be linked to openness to experience and extroversion, though novelty seeking emphasizes stimulation and sensory enjoyment. Harm avoidance could be associated with neuroticism, but harm avoidance is more focused on a biological tendency to avoid discomfort. Reward dependence corresponds to agreeableness and extraversion, but it focuses more on attachment and dependency. Persistence and self-directedness overlap with conscientiousness, though the TCI places more emphasis on self-regulation and goal setting. Cooperativeness is linked to agreeableness. Finally, self-transcendence relates in some ways to openness to experience, but it should be noted that the spiritual dimension of self-transcendence is not an explicit part of the Big-5 model.

### Study Aim

The aim of this study was to explore the association between sexual orientation and personality in a community sample of Swedish adolescents. Sexual orientation was assessed based on self-report sexual attraction, which is the recommended measure for sexual orientation in adolescence (Austin et al., 2007) and personality was measured with J-TCI (Boson et al., 2018; Luby et al., 1999). Previous research on adults (Allen & Robson, 2020) have demonstrated that homosexual and bisexual people have elevated levels of openness to experience, a facet overlapping with the TCI dimension novelty seeking. Prior studies have also found that adult asexual individuals differ from heterosexuals in that they display lower levels of extroversion, agreeableness, and conscientiousness, and higher levels of openness to experience and neuroticism (Greaves et al., 2021). Compared to other sexual minority groups, asexuals were less extraverted and agreeable. Based on this research, our primary hypothesis (H1) was that there are differences between sexual orientation groups in terms of personality dimensions among adolescents. Given the lack of prior studies on adolescent samples and studies using J-TCI to measure personality, the analyses regarding what aspects of personality would be linked to specific sexual orientations were mostly exploratory. However, based on the gender shift theory predictions (Lippa, 2005) and previous empirical findings on adolescent and adult samples (Allen & Robson, 2020; Allen & Walter, 2018; Xu et al., 2022), our secondary hypothesis were that homosexual girls score lower on harm avoidance than heterosexual girls (H2a), homosexual boys score higher on harm avoidance than heterosexual boys (H2b), and that bisexual adolescents score in-between heterosexual and homosexual peers on harm avoidance (H2c). Based on the same premises, we ran the analyses separately by gender.

## Method

### Participants and Procedure

The study was based on data from the program Longitudinal Research on Development In Adolescence (LoRDIA), which from 2013 to 2019 followed 1885 adolescents from the ages 12 and 13 years to 18 years. All 2150 students in two year-cohorts (i.e., born in 2000 or 2001) from four small- and middle-sized municipalities (10,000 to 38,000 inhabitants) in southern Sweden were invited. The unemployment rate, annual income, educational level, and proportion of first-generation immigrants across the four municipalities were close to the national means (Kapetanovic et al., 2020). Of all 2150 adolescents invited, 1885 (88%) accepted with parental consent to be part of the longitudinal research, thus forming the total LoRDIA population. Those who opted out showed no differences in demography (gender and immigration status) or school performance (attendance and academic achievements) (Kapetanovic et al., 2020).

On a yearly basis, comprehensive questionnaires were collected in classrooms by the research team starting when the students were in Grade 6 or 7 (Wave 1) up to Grade 11 at the age of 17 (Wave 5). Before the data collection of Wave 1 started, students and their parents were informed about the research program, its confidentiality, and the voluntary basis of participation. The information letter was translated into their original 32 home languages other than Swedish and sent to both parents in case of separate living. The students were given the same information adapted to their age. Parents and students had the opportunity to decline consent for the students’ participation.

This study used J-TCI (Boson et al., 2018; Luby et al., 1999), which was distributed in Wave 2 (*n* = 1459), when the students were 13 and 14 years old. Questions on sexual attraction necessary for constructing the sexual orientation categories were provided in Waves 3–5, i.e., all times later than J-TCI. In Wave 3 (*n* = 1321) participants were 14 or 15 years. Wave 4 (*n* = 726) was addressed only to the younger cohort when they were 15 years. Sexual orientation data were extracted from both Waves 3 and 4 at 15 years for the forming years of mid-adolescence. Wave 5 (*n* = 949) took place in upper secondary school when participants were 17 years and provides data for late adolescence when sexual orientation was more stabilized.

To handle some fluidity in sexual attraction from mid-adolescence to late adolescence and to optimize the use of data for this study, we based the variable sexual orientation on the latest available data on sexual attraction in each individual case, i.e., it is first based on Wave 5 (at the age of 17) and in case of missing data, these are imputed from Wave 4 or Wave 3, when the person was 15 years old.

Sexual orientation was in this way constructed for 1322 adolescents (girls *n* = 663, boys *n* = 659). To analyze their personality based on J-TCI, we chose those who had complete sets of all seven J-TCI scales from Wave 2. Combining sexual orientation with J-TCI then gave the analytical sample for this study (total *N* = 1195; girls *n* = 629, boys *n* = 566).

### Measures

Legal gender (male/female) was based on the personal ID-number given at birth or when given a residence permit, taken from tax records.

Sexual orientation was addressed in Waves 3–5 with the following two questions on attraction: “Some are emotionally and sexually attracted to people of the opposite sex and some to people of the same sex. How do you feel? (1) To some person of the opposite sex? (2) To some person of the same sex?” Both questions were responded to by one of the following alternatives: (a) Not at all attracted emotionally and sexually, (b) Somewhat attracted emotionally and sexually, c) Strongly attracted emotionally and sexually. Based on these two questions, the following four groups of sexual orientation were created: Adolescents who were: asexual (not at all attracted, neither to same nor to opposite sex); homosexual (somewhat or strongly attracted to same sex, not at all to opposite sex); heterosexual (somewhat or strongly attracted to opposite sex, not at all to same sex); and bisexual (somewhat or strongly attracted both to same and opposite sex).

The categorization constructed in this way was in total agreement with the categorization from Wave 5 of 17-year-olds (к = 1.0; *n* = 923), and in substantial agreement with the combined Wave 3–4 categorization at age 15 (к = 0.653; *n* = 1322; Absolute agreement = 0.883).

The Junior version of the Temperament and Character Inventory (J-TCI, Boson et al., 2018; Luby et al., 1999) was used in Wave 2 and consists of 105 questions used to form the 7 scales. They were formulated as statements about one's own person to be answered with “true” or “false” based on how the responder usually acts and feels. Scales were constructed for each case provided that at least 75% of scale items were answered and with imputation for missing values based on mean values of the case replies on scale items. The four Temperament scales from J-TCI are:

Novelty Seeking (NS) is based on 18 items capturing what starts behaviors, i.e., impulsivity, propensity for excitation, extravagance. Those who are high on NS have these characteristics more, while those that are low on NS tend to be more rigid, dislike rapid change, are more reserved and want more structure. Example item: “Sometimes even little things make me lose my temper” NS had a mean of 7.788 (*SD* = 3.398) and Cronbach´s alpha (α) was 0.69.

Harm Avoidance (HA) is based on 22 items capturing what inhibits behaviors, i.e., anxiety, fear of the unknown, shyness in front of strangers, and asthenia. Those who are high on HA tend to inhibit more behaviors out of anxiety, fear, shyness etc. The opposite is unlimited optimism, security in most situations, outgoing, and full of vigor. Example item: “I worry that bad things will happen”. The mean is 6.930 (*SD* = 4.408) and α was 0.82.

Reward Dependence (RD) is based on 9 items capturing traits that tend to maintain behaviors by tuning in with others. These include sensitivity to others, the need for attachment and belonging. The opposite is coldness, strong need for privacy, and independence from others. Example item: “I usually cry when I see sad movies”. The mean is 4.447 (*SD* = 2.078) and α was 0.58.

Persistence (PE) is based on 6 items capturing traits that maintain behaviors, but independent of others, such as stubbornness, enterprise, and perfectionism. The opposite is fickleness, getting bored easily, and being careless. Example item: “I try harder than other kids in school (spend more time on homework, practicing sports or instruments, etc.)”. The mean is 3.494 (*SD* = 1.379) and α was 0.31, i.e., very weak.

There are three scales on character:

Self-directedness (SD) is based on 20 items, which is about self-strength, ability to exercise self-control, take responsibility for one’s acts instead of blaming, ability to formulate goals for oneself, having access to one's strengths, self-esteem and self-acceptance, and to be congruent with one's intentions. Example item: “I usually know which goals I want to set for myself (learning new skills, getting good grades, meeting new people)”. It has a mean of 13.892 (*SD* = 3.482) and α was 0.73.

Cooperativeness (CO) is based on 20 items about abilities that make it easier for the person to work with others, e.g., tolerance of differences, being empathic and supportive, able to forgive and accept shortcomings of others, and having integrated principles of how to behave toward each other based on a notion of what is generally right, rather than to gain benefits, engage in revenge, or just pay back. Example item: “I usually like other kids even when they are very different from me”. The mean was 14.791 (*SD* = 3.161) and α was 0.69.

Self-transcendence (ST) is based on 10 items on the ability to get absorbed and to see oneself as part of something more. This includes feeling "flow" in a creative process, ability to identify with something greater (nature, spiritual values, culture, politics, etc.), as well as spiritual acceptance, e.g., experiencing the force of what unites, which can sometimes turn into mysticism and magical thinking. Example item: “I sometimes feel like living things are really connected”. The mean was 3.996 (*SD* = 3.396) and α was 0.71.

Substantial empirical support for the validity and reliability of the adult version of TCI was shown by Cloninger et al. (1993). The Junior version (J-TCI) is shorter, less than a third of the adult TCI. It gives the four temperament scales and the three character scales but does not have the subscales provided in the adult TCI. Luby et al. (1999) examined psychometric properties of J-TCI when used in children aged 9–12, and showed that scales were largely independent, with distributions similar to the adult TCI, and with acceptable Cronbach’s alpha except for RD2 (now Persistence). They also showed that J-TCI had expected gender differences and expected correlations of scales in relation to age, school performance (various aspects) and mental healthcare experiences. The confirmatory factor analysis, however, had low fit. Boson et al. (2018) investigated psychometric properties in 1046 early adolescents (12–14 years old) and congruence with caretaker reports (*n* = 481). They too found acceptable internal consistencies, except for Persistence, and expected gender differences. Inconsistencies in the correlation pattern between caretakers and children were mainly on the magnitude level but with a similar pattern. Concordance between caregivers’ reports and children’s self-reports were modest. Comparable findings on the J-TCI were published by Andriola et al. (2012), Lyoo et al. (2004), and Vangberg et al. (2013).

### Data Analysis

Relations between the four sexual orientation groups and the personality scales were studied based on the latest data collected regarding sexual orientation. For most individuals, this was age 17. The one-way analysis of variance (ANOVA) was used as a first step to find out if there were group differences in the J-TCI dimensions with Eta square (η^2^) as omnibus effect size. The following rule of thumb was used to interpret Eta square: η^2^ > 0.01 is a small effect, η^2^ > 0.06 is medium and η^2^ > 0.14 is large. In addition, post hoc power analyses were conducted to facilitate the interpretation of non-significant results (Onwuegbuzie & Leech, 2004).

We followed up with using Student’s *t* to examine the differences between pairs of groups, with Levene´s test for checking equal variance. Student’s *t* is presented with significance of difference, calculated dependent on variance being equal or not equal, and with Cohen’s *d* as effect size of the specific association. The following interpretation of Cohen’s d was used: *d* > 0.2 is a small effect, *d* > 0.5 is medium, *d* > 0.8 is a large effect. The follow-up comparisons were only performed in the cases where the omnibus test showed significance. Since the distribution on sexual orientation groups differed between genders, all analyses were gender separated.

## Results

Table 1 presents the relations between sexual orientation and personality dimensions, separately for girls and boys by providing means and standard deviations of all J-TCI scales for all sexual orientation groups. ANOVA presents omnibus statistical significance of difference as well as effect size (η^2^). For girls, the J-TCI dimensions NS, HA, RD, SD, and ST showed small and statistically significant effects. For boys, HA, PE, SD and CO showed small effects, although only HA was statistically significant. We remind the reader that the assumption of equal variances was not always met, which brings us to Table 2 which presents the results of the Student’s *t* analyses, identifying which sexual orientation groups showed statistically significant differences, as well as the effect size of those differences, Cohen’s *d*.

**Table 1:** Means (SD) of the TCI dimensions in four sexual orientation groups, with one way ANOVA showing associations between personality (J-TCI scales) and sexual orientation in adolescence, separately for girls and boys, as well as effect size (η^2^) of each association

J-TCI	Asexual (A)	Heterosexual (HE)	Homosexual (HO)	Bisexual (BI)	ANOVA (a)			Effect size	Power (*b*)
	Girls (total *n* = 629)				*F*	*df*	*p*	η^2^	1-β
	*n* = 17	*n* = 475	*n* = 28	*n* = 109					
Novelty seeking	5.08 (2.55)	7.28 (3.36)	7.54 (3.25)	7.96 (3.48)	3.877	628	0.009	0.018	0.91
Harm Avoidance	6.63 (2.98)	7.52 (4.23)	8.67 (5.20)	9.23 (4.67)	5.422	628	0.001	0.025	0.95
Reward Dependence	5.62 (1.95)	5.22 (1.99)	4.46 (1.95)	4.30 (2.20)	7.337	628	< 0.001	0.034	0.99
Persistence	4.00 (1.00)	3.60 (1.37)	3.36 (1.50)	3.37 (1.43)	1.564	628	0.197	0.007	0.47
Self-directedness	14.68 (2.97)	14.29 (3.39)	13.16 (3.90)	12.99 (3.56)	5.033	628	0.002	0.024	0.91
Cooperativeness	15.37 (2.68)	15.61 (2.79)	14.58 (3.31)	15.23 (2.83)	1.557	628	0.199	0.007	0.40
Self-transcendence	3.74 (2.39)	3.78 (2.34)	4.37 (2.27)	5.05 (2.59)	8.698	628	< 0.001	0.040	0.99

J-TCI	Asexual (A)	Heterosexual (HE)	Homosexual (HO)	Bisexual (BI)	ANOVA (a)			Effect size	Power (*b*)
	Boys (total *n* = 566)				*F*	*df*	*p*	η^2^	1-β
	*n* = 24	*n* = 501	*n* = 15	*n* = 26					
Novelty seeking	7.41 (3.02)	7.99 (3.37)	9.44 (4.12)	8.65 (3.35)	1.454	565	0.226	0.008	0.37
Harm Avoidance	6.12 (3.62)	5.79 (4.11)	5.75 (3.84)	8.84 (5.48)	4.449	565	0.004	0.023	0.90
Reward Dependence	4.02 (1.72)	3.83 (1.88)	3.50 (2.40)	3.43 (2.11)	0.600	565	0.615	0.003	0.16
Persistence	3.70 (1.29)	3.51 (1.40)	3.07 (1.16)	2.92 (1.16)	1.161	565	0.092	0.011	0.64
Self-directedness	13.61 (3.48)	14.10 (3.40)	13.38 (3.57)	12.54 (3.66)	1.969	565	0.117	0.010	0.49
Cooperativeness	13.57 (2.80)	14.38 (3.29)	12.91 (2.53)	13.28 (3.41)	2.205	565	0.087	0.012	0.69
Self-transcendence	4.10 (2.63)	3.83 (2.30)	4.00 (2.45)	4.23 (2.61)	0.345	565	0.792	0.002	0.11

^a^Note that the assumption of equal variances is not always fulfilled

^b^Power calculated post hoc in G*Power based on actual group sizes and means of each group

**Table 2:** Post-hoc comparisons of sexual orientation group means for J-TCI scales of personality for girls and boys, tested with Student’s *t* for pairs of groups. Mean differences with standard error, statistical significance and effect size (Cohen’s *d*)

		Girls				Boys			
J-TCI	Comparison	*M*^c^	*SE*	*p*	Cohen’s *d*	*M*^c^	*SE*	*p*	Cohen’s *d*
Novelty seeking	A vs. HE^a^	− 2.19	0.638	0.003	0.66				
	A vs. HO^a^	− 2.46	0.924	0.011	0.82				
	A vs. BI^a^	− 2.88	0.703	< 0.001	0.85				
	HE vs. HO	− 0.27	0.653	0.683	0.08				
	HE vs. BI	− 0.68	0.360	0.058	0.20				
	HO vs. BI	− 0.42	0.728	0.570	0.12				
Harm avoidance	A vs. HE	− 0.88	1.035	0.395	0.21	0.33	0.855	0.703	0.08
	A vs. HO^a^	− 2.03	1.221	0.103	0.45	0.37	1.220	0.763	0.10
	A vs. BI^ab^	− 2.60	0.851	0.005	0.58	− 2.72	1.304	0.043	0.58
	HE vs. HO	− 1.15	0.834	0.168	0.27	0.04	1.075	0.967	0.01
	HE vs. BI^b^	− 1.71	0.458	< 0.001	0.40	− 3.05	1.090	0.010	0.73
	HO vs. BI	− 0.56	1.012	0.578	0.12	− 3.09	1.606	0.062	0.62
Reward dependence	A vs. HE	0.40	0.491	0.417	0.20				
	A vs. HO	1.16	0.600	0.060	0.59				
	A vs. BI	1.32	0.566	0.021	0.61				
	HE vs. HO	0.76	0.387	0.050	0.38				
	HE vs. BI	0.92	0.216	< 0.001	0.45				
	HO vs. BI	0.16	0.456	0.722	0.08				
Self-directedness	A vs. HE	0.40	0.833	0.634	0.12				
	A vs. HO^a^	1.52	1.030	0.147	0.43				
	A vs. BI	1.70	0.911	0.065	0.49				
	HE vs. HO	1.13	0.665	0.091	0.33				
	HE vs. BI	1.30	0.363	< 0.001	0.38				
	HO vs. BI	0.17	0.770	0.824	0.05				
Self-transcendence	A vs. HE	− 0.04	0.577	0.942	0.02				
	A vs. HO	− 0.63	0.712	0.384	0.27				
	A vs. BI	− 1.31	0.668	0.051	0.51				
	HE vs. HO	− 0.58	0.454	0.199	0.25				
	HE vs. BI	− 1.27	0.253	< 0.001	0.53				
	HO vs. BI	− 0.69	0.535	0.201	0.27				

A = Asexual; HE = Heterosexual; HO = Homosexual; BI = Bisexual; *M* = Mean Difference; *SE* = Standard Error; *p* = Significance based on Student’s *t*; ^a^Equal variances not assumed for girls; ^b^Equal variances not assumed for boys; ^c^A minus indicates that the first group has the lower value

More group differences were found among girls than among boys. Adolescent girls in the asexual group scored significantly lower than all other three groups on NS, lower than bisexual girls on HA, and higher than bisexuals on RD. They furthermore tended to score higher than homosexual girls on RD (*p* = 0.06), higher than bisexuals on SD (*p* = 0.07) and lower than bisexuals on ST (*p* = 0.05).

Moreover, bisexual girls compared to heterosexual girls scored higher on HA and ST, lower on RD and SD, and tended to score higher on NS (*p* = 0.06). In addition to the already mentioned differences compared to asexuals on NS and RD, homosexual girls tended to score lower than heterosexual girls on RD (*p* = 0.05). The effect sizes for girls ranged from small to large with the findings concerning NS being the largest. The comparisons between asexual girls on the one hand and bisexual and homosexual girls on the other yielded the largest effect sizes of all comparisons.

Boys in the bisexual group reported higher levels of HA compared to asexuals and heterosexuals and tended also to score higher than homosexuals (*p* = 0.06).

## Discussion

Adolescence is a time of sexual development and maturation, but also of personality development. This study was designed to explore and compare personality dimensions among adolescents in different sexual orientation groups using the J-TCI (Boson et al., 2018; Luby et al., 1999). To our knowledge, this is the first time such a study has been presented in the literature. In short, using a community-based sample of adolescents, the findings revealed several temperament and character differences between sexual orientation groups among girls, with several of the group differences being large, and fewer differences between sexual orientation groups among boys. Accordingly, the present study offers a significant, incremental contribution to the field.

Our first hypothesis, that there are significant personality differences between sexual orientation groups, was supported, for both boys and girls. However, the only statistically significant finding for boys was that the group of bisexuals scored higher on one of the temperaments, harm avoidance (anxiety, fear of the unknown, shyness in front of strangers, and asthenia), than heterosexuals and asexuals, with effect sizes being medium in size. This finding contrasts our secondary hypothesis (H2b) and the gender shift theory suggesting that bisexual individuals are intermediate between heterosexual and homosexual individuals (Lippa, 2005). At the same time, our finding is partly in line with the secondary hypothesis H2c and the findings by Xu et al. (2022), who found higher levels of neuroticism (a trait strongly associated to harm avoidance) among bisexual compared to heterosexual youth. It is also in line with some prior research on adults (Allen & Robson, 2020; Wang et al., 2014). Moreover, bisexual adults have been found to experience more identity confusion than other groups (Balsam & Mohr, 2007). Identity confusion, in turn, has been linked with outcomes strongly related to neuroticism and harm avoidance in adolescence (e.g., anxiety, Potterton et al., 2022). Given that adolescence is a time of sexual, personality, and identity development, potential links among the three constructs in adolescence should be explored further in future research.

We found more statistically significant associations between sexual orientation and personality dimensions—pertaining to both temperament and character—for girls. The “gendered” nature of the associations corroborates what has been demonstrated previously regarding health behavior outcomes, including alcohol and drug use, where effects of sexual orientation have been found to be stronger for young women than for young men (Coulter et al., 2018; Gerdner et al., 2024; Roxburgh et al., 2016). Why effects of sexual orientation seem to be stronger for girls than boys across a spectrum of outcomes needs to be explored in future studies. From a social-environmental perspective (Klimstra et al., 2009), it could be argued that traditional gender norms often dictate stricter standards of behavior for girls and women compared to boys and men. Non-heterosexual girls may experience more conflict with these norms, leading to greater psychological distress and other negative outcomes. From a psychological perspective, it could be proposed that boys and girls may employ different coping strategies and mechanisms in response to societal pressures related to sexual orientation, which could affect the manifestation and impact of outcomes, including personality and health. These explanations, however, may be more valid for differences concerning the character rather than the temperament dimensions, given that the latter have been demonstrated to have high heritability in behavior genetic research (Zwir et al., 2021).

Among the current findings pertaining to girls, there were significant associations between sexual orientation and four of the personality dimensions: novelty seeking, harm avoidance, reward dependence, and self-transcendence. Several notable observations emerged. First, bisexual girls’ personalities differed the most from the other groups of girls except for from homosexual girls. In addition to scoring higher on harm avoidance, they also scored higher on novelty seeking and self-transcendence, and lower on reward dependence and self-directedness. Thus, among both genders, the group of bisexual individuals differ significantly from the other groups of adolescents except for the group of homosexuals. It is interesting to note that the overall personality profile of bisexual girls—high scores on novelty seeking, and harm avoidance, and low scores on reward dependence and self-directedness—resembles that of individuals with emotional instable personality (Cloninger, 2002). In addition, they score high on self-transcendence which usually contributes color to a so-called mature personality, but in combination with low self-directedness instead may increase vulnerability to clinical and sub-clinical psychotic experiences or schizotypal traits as well as to suicidality (Cortés et al., 2009; Mamah et al., 2020; Nitzburg et al., 2014; Yang et al., 2021).

In line with what was the case for boys, these findings for girls contrast with previous research based on the gender shift theory (Lippa, 2005) and the secondary hypotheses (i.e., H2a and H2c), where bisexuals are assumed to be intermediate in terms of personality gender typicality. Consequently, while the gender-shift hypothesis might provide a useful framework for understanding personality differences across sexual orientation groups, our findings indicate that it does not fully account for all observed patterns. For example, consistent with the hypothesis, homosexual females scored intermediate between heterosexual males and females on novelty seeking. However, bisexual females exhibited even higher novelty seeking scores than both heterosexual and homosexual females, making them more "male-shifted" in this trait. Similarly, in harm avoidance, bisexual males—not homosexual males—showed a notable deviation from heterosexual males, suggesting a pattern that is not entirely predicted by the gender-shift framework. These findings align with prior research indicating that bisexual individuals often differ from both heterosexual and homosexual individuals in meaningful ways (Alley et al., 2024; Diamond & Alley, 2019; Semenyna et al., 2024). One potential explanation is that bisexual individuals may experience greater biopsychosocial instability, such as heightened sensitivity to reward processing or adverse childhood experiences, which in turn could shape personality development (Alley et al., 2024). Another possibility is that bisexuality, as a distinct orientation, involves unique developmental pathways that interact with personality traits in ways that are not adequately captured by a strictly gender-typicality-based model.

A second notable observation for girls in the current study was that the group of asexual girls scored significantly lower on novelty seeking than all other girls. The overall personality profile with low novelty seeking, low harm avoidance, and high reward dependence in combination with normal levels of self-directedness and cooperativeness and low self-transcendence describe individuals who are stable, reliable, content with life as is. Such individuals are not looking for excitement and they suffer less from anxiety or negative affect. They function among others and thus seem to cope with life well without sex as part of it. Other scholars have drawn similar conclusions about the group of asexual adults, that they are not to be regarded as psychologically or socially indifferent, but that they may be more cautious in relation to other people (Greaves et al., 2021).

A recent study based on structured diagnostic interviews (Gerdner et al., 2024) found that bisexuals of both genders as well as homosexual girls had more mental health problems than other sexual orientation groups, while asexuals of both genders were in the opposite end, with few mental health problems. Similarly, bisexual adult women have been identified to be a particular risk of co-occurring psychological distress and substance use (Bränström & Pachankis, 2018). The findings of the current study reveal that the very same groups of adolescents also differ in terms of personality dimensions, and in the expected directions, since high scores on harm avoidance is known to be related to emotional problems, and high scores on novelty seeking are related to extrovert behavioral problems, both more so when combined with low scores on self-directedness indicating more immature personality problems (Fassino et al., 2013; Svrakic et al., 2002). According to da Costa et al. (2020), harm avoidance is a mediator between early stress from childhood maltreatment and mental health problems in adulthood. Correspondingly, harm avoidance and other personality dimensions may be mediators in the relation between minority stress, often theorized as a major cause of more mental health problems in sexual minorities (Hoy-Ellis, 2023), and mental health problems.

The observed associations between personality dimensions and sexual orientation development may be underpinned by several plausible mechanisms, which warrant further exploration. One potential explanation involves common hormonal and genetic influences that shape both personality and sexual orientation during early development. For instance, prenatal androgen exposure has been proposed as a mechanism influencing both the development of gendered personality traits (e.g., higher novelty seeking and lower harm avoidance) and non-heterosexual orientations, with variations in hormonal exposure potentially creating patterns of sex differences observed in these domains (Aguilar, 2023; Luoto, 2020). Moreover, social and environmental influences, such as societal expectations and discrimination, may shape personality development differently for girls and boys based on their sexual orientation. Biological mechanisms, including hormonal influences, might also play a role. Additionally, distinct coping strategies and identity formation processes could impact traits like self-directedness. Cultural norms and minority stress experiences may further contribute to these differences. For instance, higher harm avoidance in bisexual boys might reflect a response to societal challenges (Lippa, 2020). These explanations highlight the complex interplay of social, biological, and psychological factors in shaping personality traits across sexual orientation groups. Future research could examine how these mechanisms might interact to influence both personality and sexual orientation, potentially generating sex-specific patterns in their associations, moving beyond exploratory approaches to propose specific hypotheses that can better test these potential causal pathways is an important next step. Such hypotheses could focus on disentangling the relative contributions of biological and social factors, as well as their interactions, in driving the observed associations between personality and sexual orientation among adolescents.

### Limitations and Future Research Directions

This study has several limitations that should be considered when interpretating the findings and designing future studies in the field. First, the numbers of adolescents in the sexual minority groups, especially the asexual and homosexual groups, were small. Small group sizes impact power negatively, but effect sizes might still be accurate. Second, this study had a design where personality was measured only once and since all measures on sexual orientation were in later waves, we could not test directions of effects. Longitudinal studies, in which both sexual orientation and personality are measured repeatedly over the course of adolescence, are needed to determine the direction of effects between sexual orientation and personality, given that both structures show some fluidity over time (Baams & Kaufman, 2023; Bleidorn et al., 2022; Claesdotter-Knutsson et al., in press). Third, we did not explore possible explanations of the identified relations. Future research is needed to explore mechanisms and processes linking sexual orientation and personality as well as moderating conditions (e.g., context). Fourth, we measured only one aspect of sexual orientation (i.e., emotional and sexual attraction). Whereas this is the recommended measure choice in the current study population age (Austin et al., 2007), other aspects of sexual orientation (i.e., identity and behavior) could be differently associated with personality dimensions, especially given that the three aspects do not fully overlap on the individual level (Priebe & Svedin, 2013). In line with Baams and Kaufman (2023), we call for future research that tap into the full range of sexual orientation aspects. Finally, more research is needed to determine at what age associations between sexual orientation and personality emerge. Meta research has established such associations among adults (Allen & Robson, 2020), which means that we now have evidence to suggest that the associations exist in both adulthood and adolescence, but the body of research on younger populations needs to be expanded to understand when the associations first appear.

### Conclusions

To our knowledge, this is the first study to explore relations between sexual orientation and personality as measured by temperament and character in adolescence. The study revealed several significant differences in both temperament and character dimensions of personality between sexual orientation groups among girls, and to a lesser extent among boys. In doing so, the current findings offer an interesting window into the lives of youth with different sexual orientations and provide cues to some of the previously well-established associations between sexual orientation on the one hand and psychosocial and mental health outcomes on the other (Gerdner et al., 2024; Gmelin et al., 2022; Plöderl & Tremblay, 2015; Wittgens et al., 2022). At the same time, it is important to note that the effects sizes were generally, but not always, small. Far from all individuals with the same sexual orientation shared the same temperament and character traits. Because the association between sexual orientation and personality is a complex and multifaceted topic, the current findings should be regarded as preliminary until they have been replicated in other samples. Future research could employ larger and more diverse samples, utilize more comprehensive measurement tools, and account for social and cultural influences to better understand the association between sexual orientation and personality among developing adolescents.

## Data Availability

The data sets generated and/or analysed during the current study are not publicly available due to ethical regulations regarding the LoRDIA program. Swedish data protection laws restrict sharing of the data. According to these laws, the data can be made available for research projects with pre-defined and ethically approved research questions. Requests to use the LoRDIA data should be directed to the steering committee of the LoRDIA program through the PI of The Gothenland Millenium Cohort, Professor Tina M. Olsson at Jönköping University, Sweden. Data can be made available after the steering committee has reviewed the request and the Swedish Ethical Review Authority has approved the research questions.
